# Harvest Stage‐Dependent Variation in Essential Oil Chemotype and Agronomic Yield of Parsley (*Petroselinum crispum* (Mill.) Fuss)

**DOI:** 10.1002/fsn3.72235

**Published:** 2026-08-07

**Authors:** Ayşe Betül Avci, Özlem Alan, Merve Göre, Bintuğ Öztürk

**Affiliations:** ^1^ Department of Plant and Animal Production, Ödemiş Vocational School Ege University İzmir Turkey; ^2^ Department of Pharmaceutical Botany, Faculty of Pharmacy Ege University İzmir Turkey

**Keywords:** apiol, chemotype, essential oil, GC–MS, harvest time, *Petroselinum crispum*, phenylpropanoid

## Abstract

Harvest timing is a critical agronomic factor influencing both yield and phytochemical quality in aromatic plants. This study evaluated the effect of different harvest stages on biomass production, essential oil (EO) yield, chemical composition, and chemotype variation in parsley ( *Petroselinum crispum* (Mill.) Fuss). Field experiments were conducted under Mediterranean conditions (Ödemiş/İzmir, Türkiye), and plants were harvested at four distinct developmental stages (Pet‐1 to Pet‐4, May to September). Essential oils were extracted by hydrodistillation and analyzed using GC–MS, while multivariate analyses (HCA and PCA) were applied to assess compositional variation. Biomass yield increased progressively with delayed harvest, whereas EO content showed a non‐linear pattern, peaking at the second harvest (0.73%) and declining thereafter, indicating a trade‐off between yield and oil concentration. GC–MS analysis identified 55 volatile compounds across all harvest stages (identification rate: 94.47%–96.47%). Early harvests were characterized by higher proportions of monoterpene hydrocarbons (particularly β‐phellandrene), while later stages showed increased accumulation of phenylpropanoids (apiol reaching 43.69% at Pet‐4) and, notably, sesquiterpenes (caryophyllene (Z): 20.86% at Pet‐3, the accumulation of which may be associated with prevailing summer conditions). Principal component analysis (PC1 = 62.1%, PC2 = 30.6%; cumulative = 92.7%) clearly separated harvest stages based on chemical profiles, supporting the presence of two distinct chemotypes: a monoterpene‐balanced type (Chemotype A: Pet‐1, Pet‐2) and a phenylpropanoid‐dominant type (Chemotype B: Pet‐3, Pet‐4). These findings demonstrate that harvest timing plays a decisive role in shaping both EO composition and chemotype in parsley. Moreover, the observed variation in key constituents, particularly caryophyllene and myristicin, may influence the functional properties of the oil, as these compounds are associated with antioxidant and antimicrobial activities. Optimizing harvest stage is therefore essential not only for maximizing biomass yield but also for improving the qualitative and potential bioactive value of parsley essential oil under specific environmental conditions.

## Graphical abstract

Parsley ( *Petroselinum crispum* ) was harvested at four ontogenetic stages (Pet‐1 to Pet‐4; May–September) under Mediterranean field conditions. Harvest timing significantly affected both agronomic yield and essential oil (EO) composition. Biomass yield peaked at Pet‐4 (10,076 kg da^−1^); EO content was highest at Pet‐2 (0.70%). GC–MS identified 55 volatile compounds. Early harvests were dominated by monoterpene hydrocarbons (β‐phellandrene), while later stages showed progressive accumulation of phenylpropanoids (apiol: 43.69% at Pet‐4). Two chemotypes were resolved by HCA and PCA (92.7% variance explained): a monoterpene‐balanced type (Pet‐1/2) and a phenylpropanoid‐dominant type (Pet‐3/4). Harvest stage is a key determinant of EO chemotype in parsley.

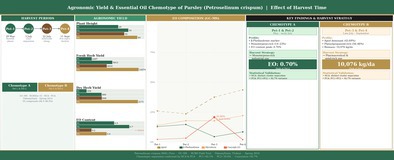

## 1 Introduction

Parsley ( *Petroselinum crispum* (Mill.) Fuss) is an important biennial medicinal and aromatic plant belonging to the Apiaceae (Umbelliferae) family, which is widely consumed worldwide in both fresh and dry forms (Subaş et al. 2024; Ahmed et al. 2025). Originating from the Mediterranean basin, this plant is recognized today as a valuable raw material in the pharmaceutical, perfume, and cosmetic industries, in addition to its use as a flavoring agent and garnish in culinary applications (Bahramsoltani et al. 2024; Lopez et al. 1999). The biological and economic value of parsley stems from its content of vitamins (A, C, K), minerals, and, most importantly, its pharmacologically active secondary metabolites (Ahmed et al. 2025; Subaş et al. 2024). Essential oils obtained from the aerial parts and seeds of the plant possess a wide therapeutic spectrum, including antioxidant, anti‐inflammatory, antibacterial, hepatoprotective, and hypoglycemic activities (Zhang et al. 2006; Nouioura et al. 2024). Information and standards related to the medicinal effects of parsley are included in several pharmacopeias and international medicinal plant monographs (Commission E; British Herbal Pharmacopeia (BHP); British Herbal Compendium (BHC)).

The characteristic aroma and medicinal efficacy of parsley essential oil depend on the relative proportions of key components, primarily myristicin, apiol, β‐phellandrene, and 1,3,8‐p‐menthatriene (Petropoulos et al. 2004; Gruszecki and Walasek‐Janusz 2022). However, the synthesis and accumulation of these components are not constant and are directly influenced by parameters such as genetic structure, climatic factors, and harvest management (Figueiredo et al. 2008; Said‐Al Ahl et al. 2016).

Among these factors, harvest time (or the plant's developmental stage) is one of the most critical elements determining EO yield and quality (Petropoulos et al. 2004; Said‐Al Ahl et al. 2016). Studies in the literature indicate that the distribution of volatile components varies over time throughout the growth cycle of parsley, and different vegetative stages result in distinct aroma profiles (El‐Zaeddi et al. 2020; Petropoulos et al. 2004). Furthermore, as parsley is a crop suitable for consecutive harvests (multi‐cut), the environmental conditions at each harvest period (temperature, day length, etc.) create different dynamics regarding biomass yield and oil composition (Abdalla and Elriah 2024; Sany et al. 2022).

Establishing optimal harvest strategies is essential both to maximize fresh herb yield per unit area and to achieve the specific chemical profile targeted for industrial purposes (Alharbi et al. 2019; Said‐Al Ahl et al. 2016). Nevertheless, there is still a need for up‐to‐date and specific data regarding the changes in yield and quality exhibited by parsley cultivars grown under different ecological conditions relative to harvest time.

The objective of this study is to determine the optimum harvest schedule for the local İzmir/Ödemiş parsley ecotype by evaluating the concurrent shifts in biomass accumulation, dry matter content, and EO profiles throughout its ontogenetic development. Furthermore, this research aims to establish a scientific framework for maximizing both industrial yield and phytochemical quality, providing actionable data for the standardization of high‐quality raw materials in the Mediterranean region.

## 2 Material and Methods

This study was conducted at the experimental field (38°12′N; 27°52′ E; 111 m elevation) and laboratories of Ege University Ödemiş Vocational School, as well as the laboratories of Toros Tarım Ticaret A.Ş. to investigate the effects of harvest stage on yield and quality parameters in parsley plants.

### 2.1 Material

#### 2.1.1 Plant Material

Parsley ( *Petroselinum crispum* ) seeds, obtained from the Balıkesir region—one of Turkey's important production centers—were used as the plant material in the study. Parsley is an important biennial aromatic plant belonging to the Apiaceae family, consumed globally in both fresh and dry forms (Subaş et al. 2024). The seeds used in the trial were selected from local populations compatible with the regional ecology.

#### 2.1.2 Soil Characteristics

Soil analysis of the trial field was conducted in the soil analysis laboratories of Toros Tarım Sanayi ve Ticaret A.Ş. The results revealed that the experimental soil has a clay loam structure with 1% organic matter content, 0.03% total salt concentration, and 2.7% lime content. With a pH value of 7.15, the soil exhibits a neutral reaction. Literature reports that parsley exhibits optimal development in well‐drained soils containing high organic matter with a pH value between 5.3 and 7.3 (Borges et al. 2016; Mirmohammadmakki et al. 2023). The analysis also indicated that the soil is poor in organic matter, which is an important consideration for crop production in the experimental area.

#### 2.1.3 Climate Data

The climate data for the study year shows seasonal temperature variations in the experimental area. The average temperature, which was 11.5°C in March, gradually increased to reach the highest value of 28.1°C in August. The highest temperature of the year was recorded as 39.7°C in August. Relative humidity values for July and August were determined as 49.9% and 53.8%, respectively. From September onwards, temperatures began to decline, decreasing to an average of 10.9°C in December. The lowest temperature values recorded throughout the year fell below zero in March, November, and December.

### 2.2 Methods

#### 2.2.1 Experimental Design and Cultural Practices

Field trials were conducted during the 2014 growing season and established in accordance with a randomized complete block design (RCBD) with three replications. Data were subjected to one‐way ANOVA using IBM SPSS Statistics (v.26.0; IBM Corp., Armonk, NY, USA). Prior to ANOVA, normality of residuals was verified using the Shapiro–Wilk test and homogeneity of variances was assessed using Levene's test; both assumptions were satisfied for all variables (*p* > 0.05). Significant differences among means (*p* ≤ 0.05) were identified using Fisher's least significant difference (LSD) test, and least significant difference (LSD) values are presented in all relevant tables. Experimental plots were planned with dimensions of 1.80 m × 3 m, totaling an area of 5.4 m^2^. Within the scope of spring sowing, seeds were sown manually on March 20, with a row spacing of 30 cm and at a rate of 1.5 g/m^2^. Prior to sowing, 15–15‐15 nitrogen‐phosphorus‐potassium (NPK) compound fertilizer was applied as a basal dressing at rates of 8 kg N, 5 kg P_2_O_5_, and 5 kg K_2_O per decare. The water requirements of the plants were met via a drip irrigation system, and weed control was performed manually throughout the duration of the trial. To combat powdery mildew observed during the spring cultivation period, two applications of a preparation containing the active ingredient Penconazole (Topas) were administered at 15‐day intervals.

#### 2.2.2 Harvest Protocol and Timing

Due to parsley being a species suitable for multi‐cut harvesting, the plants were harvested at four distinct ontogenetic stages. Pet‐1 (29 May) corresponded to the early vegetative stage (rosette phase, approximately 10 weeks after sowing); Pet‐2 (3 July) to the mid‐vegetative stage (active leaf expansion); Pet‐3 (22 July) to the late vegetative stage (onset of internode elongation, coinciding with peak summer temperatures); and Pet‐4 (11 September) to the advanced vegetative–pre‐reproductive stage (maximum canopy development). Harvesting was performed manually by cutting the plants just above the soil level. A total of four cuttings were conducted for the spring sowing on May 29, July 3, July 22, and September 11, respectively.

#### 2.2.3 Determination of Agronomic Characteristics

The following parameters were measured to determine plant yield and quality characteristics during the harvest periods. The agronomic characteristics of parsley were comprehensively examined in the study. Plant height measurements were conducted before harvesting, with the average values calculated by measuring the distance from soil level to the tip of 10 randomly selected plants from each plot. When determining fresh herb yield, all remaining plants were cut at a height of 5–7 cm from the ground after removing the edge effects in each plot, and their fresh weights were measured. To determine the drug herb ratio, 500 g fresh samples taken from each plot were dried at 35°C, and the post‐drying weight was calculated as a percentage. Drug herb yield was obtained by multiplying the fresh herb yield by the drug herb ratio and dividing the result by 100. These parameters were used as fundamental data to determine the yield potential and quality characteristics of parsley at different harvest periods.

#### 2.2.4 Quality Analysis

##### 2.2.4.1 Essential Oil Content (%)

Essential oil content was determined volumetrically using the Neo‐Clevenger apparatus on plant material dried at 35°C. Essential oil content was expressed as milliliters per 100 g (%) of dry matter and determined in three technical replicates per biological sample (Wichtl 2004). Biological replicates corresponded to the three independent field plots of the RCBD design (*n* = 3 per harvest period). EO content values reported are means of three biological replicates.

##### 2.2.4.2 Essential Oil Isolation and Chromatographic Analysis

As with some other plant species in the Apiaceae family, the composition of parsley essential oil is determined by gas chromatography–mass spectroscopy (GC–MS) analysis (Bahramsoltani et al. 2024). Thermo Scientific Trace GC Ultra gas chromatograph (Thermo Scientific Ltd., Rodano, Milan, Italy), equipped with a Thermo Scientific Focus GC flame‐ionization detector (ChromQuest 4.2.34) and a Thermo TR‐Wax fused silica capillary column (60 m–0.32 mm i.d.; film thickness, 0.25 mm) was used for GC analyses. Diluted EO sample (1/100 in n‐hexane, v/v) injection was utilized with Thermo Scientific TriPlus autosampler (Thermo Scientific Ltd.) in split mode with an inlet temperature of 200°C, split flow of 50 mL/min and split ratio of 50. Oven temperature was programmed from 70°C to 210°C at 7°C/min, and the final temperature was held for 10 min. Also, the detector temperature was 230°C, and the carrier gas (Helium) had a flow rate of 1 mL/min. The relative percentage of the EO constituents was calculated from the GC‐FID (GC with flame‐ionization detection) peak areas (Ulukanli et al. 2014). Results are expressed as relative peak area percentages, consistent with standard practice in descriptive essential oil profiling (Adams 2007; Bakkali et al. 2008; Petropoulos et al. 2004). GC–MS and GC–FID analyses were performed on pooled EO samples representative of each harvest period. Each analysis was conducted in three technical replicates, and reported values represent the mean of these replicates. Representative GC–MS chromatograms for all four harvest periods are provided as Figure S1.

EO analysis was performed using the same Thermo Scientific Trace GC Ultra gas chromatograph (Thermo Scientific Trace GC Ultra & DSQ II) couplet with a Thermo Scientific DSQ II mass detector (2.0.1 SP1), and a Thermo TR‐Wax fused silica capillary column (60 m–0.32 mm i.d.; film thickness, 0.25 mm) set at 70 eV. The chromatographic conditions adopted for GC–MS analyses were the same as for GC‐FID. The identification is based on comparing the arithmetic indices (AI), calculated using the retention times of the compounds in the essential oil and the C8–C20 n‐alkane mixture, with reference values (Adams 2007). The results were also confirmed by computer matching of mass spectra with the mass spectra data libraries in Excalibur software (Xcalibur v.2.0.7).

## 3 Results

### 3.1 Agronomic Characteristics

As a result of the research, 4 harvests were carried out in the spring planting of 2014, and the differences between harvests were found to be statistically significant in terms of plant height, fresh herb yield, dry herb yield, and EO content (Table 1). While the EO content reached its highest value in the second harvest conducted in July, the highest yields in all other parameters were obtained in the 4th and final harvest carried out in September.

**TABLE 1:** Average plant height, fresh herb yield, dry matter, dry herb yield, and EO content of parsley ( *Petroselinum crispum* ) for the experimental years.

Treatment	Plant height (cm)	Fresh herb yield (kg da^−1^)	Dry matter (%)	Dry herb yield (kg da^−1^)	EO content (%)
1st harvest	27.71c	2486.67c	14.48c	358.58c	0.53b
2nd harvest	34.33ab	5813.33b	15.66c	889.27b	0.73a
3rd harvest	31.70bc	4910.00b	18.25b	895.96b	0.23c
4th harvest	40.33a	10,076.33a	20.17a	2170.89a	0.40bc
Mean	33.52	3571.58	17.14	1078.67	0.47
Fisher's LSD	6.109	2173.297	1.904	251.59	0.019

*Note:* Values with different letters in the same row show a difference of *p* < 0.05 according to the Fisher's LSD test.

#### 3.1.1 Plant Height

**FIGURE 1:**
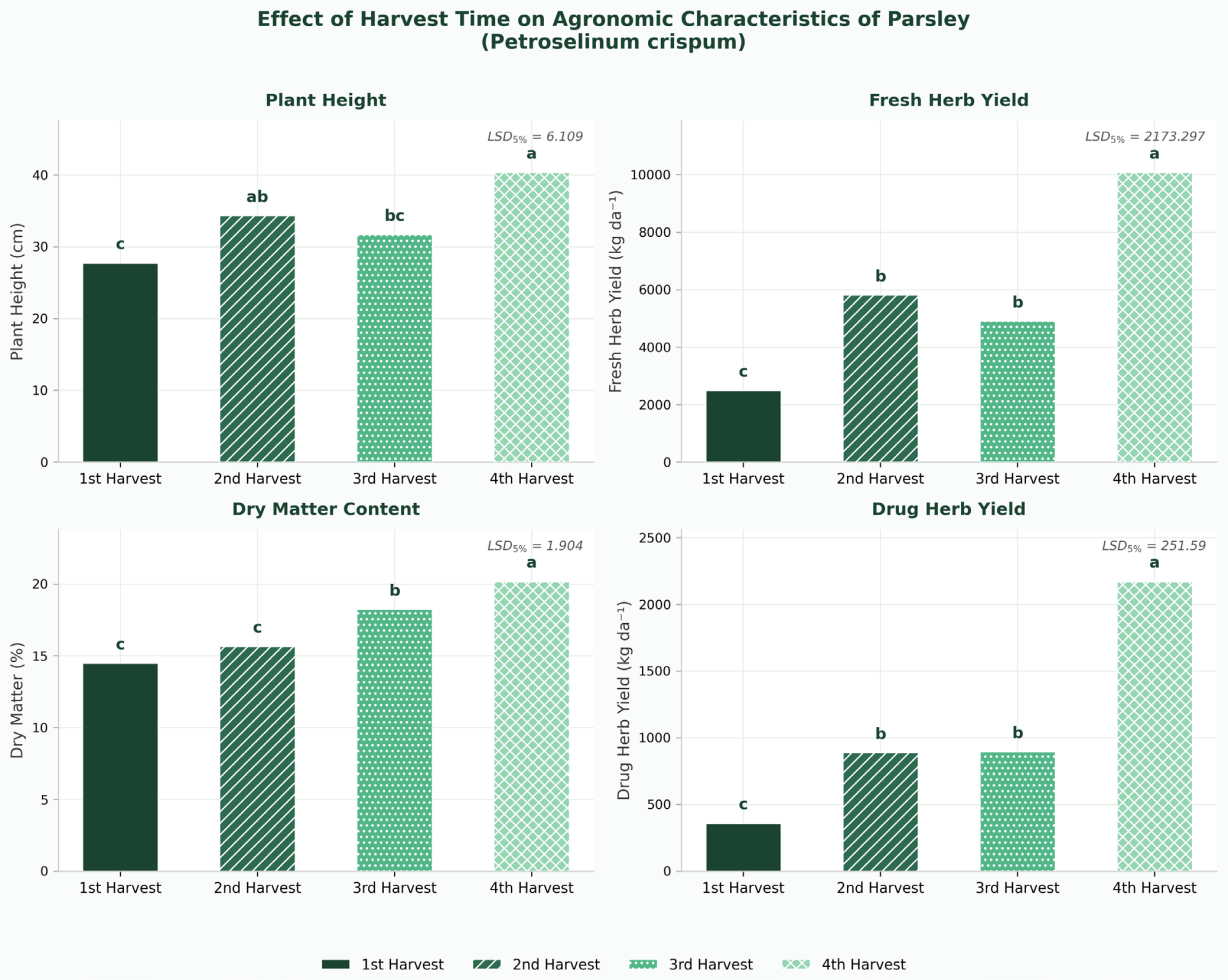
Effect of harvest time on agronomic characteristics of parsley ( *Petroselinum crispum* ) (A) plant height (cm), (B) fresh herb yield (kg da^−1^), (C) dry matter content (%), and (D) drug herb yield (kg da^−1^). Bars represent means; different letters indicate significant differences at *p* ≤ 0.05 (Fisher's LSD test). LSD values are shown within each panel.

In the study conducted with the İzmir/Ödemiş ecotype, the effect of harvest time on morphological development was found to be statistically significant (27.71–40.33 cm; +45.5%, *p* < 0.05; Figure 1A). The 4th harvest (Pet‐4), which achieved the highest plant height, confirms that the plant's vegetative growth capacity reached its maximum in the late period. The overall mean of 33.52 cm is within the reported growth range for the species (Subaş et al. 2024; Ahmed et al. 2025). The linear increase observed from the 1st harvest (27.71 cm) to the 4th harvest (40.33 cm) indicates continuous biomass accumulation throughout the vegetation period. In the literature, Said‐Al Ahl et al. (2016) support this finding by reporting that the highest height values were reached in the 3rd harvest (165th day), depending on the developmental stage (Said‐Al Ahl et al. 2016; Gruszecki and Sałata 2013). While the height values determined in this study show parallelism with those of Said‐Al Ahl et al. (2016) (11.1–43.3 cm), they are higher than the values of 11.67–14.67 cm emphasized by Aziz et al. (2013) (Said‐Al Ahl et al. 2016; Aziz et al. 2013). The slight reduction in height between the 2nd harvest (34.33 cm) and the 3rd harvest (31.70 cm) can be associated with the pressure of high summer temperatures and water stress on the plant at the end of June (Borges et al. 2016).

Parsley is extremely sensitive to water stress, and severe water deficiency can reduce the length of aerial parts from 20 cm to as low as 15.33 cm (Borges et al. 2016). This environmental stress is thought to redirect plant metabolism from height growth toward the production of defense compounds, such as the essential oil changes observed in Pet‐2 and Pet‐3 (Borges et al. 2016).

The peak value of 40.33 cm suggests adequate agronomic control of stress factors during the trial period (Shamshiri et al. 2024). Compared to values reaching up to 56.1 cm with phosphorus fertilization under Egyptian conditions (Alharbi et al. 2019), values in Ödemiş may reflect the influence of local soil characteristics and agronomic management (Alharbi et al. 2019; Borges et al. 2016).

#### 3.1.2 Fresh Herb Yield and Biomass Accumulation

A substantial increase of 305% in fresh herb yield was observed throughout the ontogenetic development of parsley, progressing from Pet‐1 to Pet‐4 (2486.67 → 10076.33 kg da^−1^, *p* < 0.05; Figure 1B). This yield, which peaked during the 4th harvest in early September (approximately 10 tons/da), indicates that the plant's biomass accumulation potential reaches its maximum as it matures (Said‐Al Ahl et al. 2016).

This high yield may reflect the adaptation of the local ecotype to regional climatic and edaphic conditions (Borges et al. 2016).

The numerical stagnation observed at the end of June (3rd harvest) parallels the yield decline among summer harvests found in studies conducted in Egypt (Sany et al. 2022). This lull is associated with the diversion of plant resources from vegetative growth to secondary metabolite production (defense mechanism) due to environmental stress caused by the high summer temperatures in Ödemiş (Borges et al. 2016; El‐Zaeddi et al. 2020; Shamshiri et al. 2024).

These yield differences likely reflect the combined effects of harvest timing and the regional adaptation of the local ecotype (Alharbi et al. 2019; Said‐Al Ahl et al. 2016).

#### 3.1.3 Dry Matter and Ontogenetic Development

During the ontogenetic development process of parsley, the dry matter content exhibited a gradual and significant increase from 14.48% to 20.17% (+39.3%, *p* < 0.05; Figure 1C). The statistical differentiation of Pet‐3 and Pet‐4 samples from Pet‐1 and Pet‐2 confirms the plant's physiological maturation process (Alharbi et al. 2019; Said‐Al Ahl et al. 2016). The increase in tissue density and the accumulation of carbohydrates and fibers as the vegetation period extends is consistent with literature findings reporting that late harvests provide higher dry weight (Said‐Al Ahl et al. 2016; Ahmed et al. 2025; Kołota and Adamczewska‐Sowińska 2012; Gruszecki and Walasek‐Janusz 2022; Antonopoulos et al. 2014).

In early stages, high water content (85.52%–84.34%) reflected active cell division, while progressive cell wall thickening and lignification increased the dry matter ratio in later stages (Zheljazkov et al. 2008; Figueiredo et al. 2008).

The strong positive correlation between dry matter and the F/M ratio (*r* = 0.99, *p* < 0.01) suggests that structural and chemical maturation progress in parallel (Petropoulos et al. 2004; Said‐Al Ahl et al. 2016). Owing to this relationship, the dry matter ratio can be utilized as an indirect indicator of EO composition: a ratio of 14%–16% indicates the monoterpene‐rich “Chemotype A,” while 18%–20% points to the phenylpropanoid‐dominant “Chemotype B” (Said‐Al Ahl et al. 2016). This increase in dry matter ratio provides economic savings by reducing drying costs, while improving product stability (Said‐Al Ahl et al. 2016).

#### 3.1.4 Dry Herb Yield

Dry herb yield of parsley ranged from 358.58 to 2170.89 kg da^−1^, with the peak yield recorded in the final harvest and the lowest in the first. Aziz et al. (2013) determined the dry herb yield in the control group to be 6.10 g/plant. The largest increase during the ontogenetic development process was observed in the dry herb yield, which surged by 505% from Pet‐1 to Pet‐4, representing an approximately 6‐fold increase (*p* < 0.05; Figure 1D). As this parameter is the product of fresh herb yield and the dry matter ratio, it reflects the cumulative effect of the increases in both variables.

The yield growth followed an exponential pattern; the 148.0% increase from Pet‐1 to Pet‐2 reflects both biomass and dry matter accumulation during the transition from the early to middle period. The minimal increase during the transition from Pet‐2 to Pet‐3 (0.8%) was due to the decrease in fresh herb yield being offset by the rising dry matter ratio. The most pronounced yield increase occurred between Pet‐3 and Pet‐4 (142.3%), peaking at 2170.89 kg da^−1^during the final harvest in early September. This upward trend is directly associated with the increase in dry matter ratio (from 14.48% to 20.17%) during the plant's maturation stage. These findings are supported by Said‐Al Ahl et al. (2016), who reported that total biomass in parsley is maximized in the 3rd and 4th harvests. This superior performance in September is attributed to the agronomic advantages of the Ödemiş region, including its prolonged sunshine duration, loamy‐sandy soil structure, and balanced pH of 7.15.

The peak dry herb yield obtained in Ödemiş (2170.89 kg da^−1^) is substantially higher than values reported in comparable studies; for example, Sany et al. (2022) reported 183–235 kg da^−1^ reported by Sany et al. (2022) for curly‐leafed parsley grown in Egypt.

These substantial differences may reflect the genetic potential of the local ecotype, the region's optimal climatic conditions, and the success of the applied agronomic techniques. The 4th harvest at the beginning of September (within the LSD margin of 251.59) has been identified as the most economically efficient period for commercial production, providing the maximum raw material output per unit area. Optimizing the harvest schedule to coincide with this peak in dry matter accumulation enhances processing efficiency while simultaneously minimizing drying costs.

#### 3.1.5 EO Content

**FIGURE 2:**
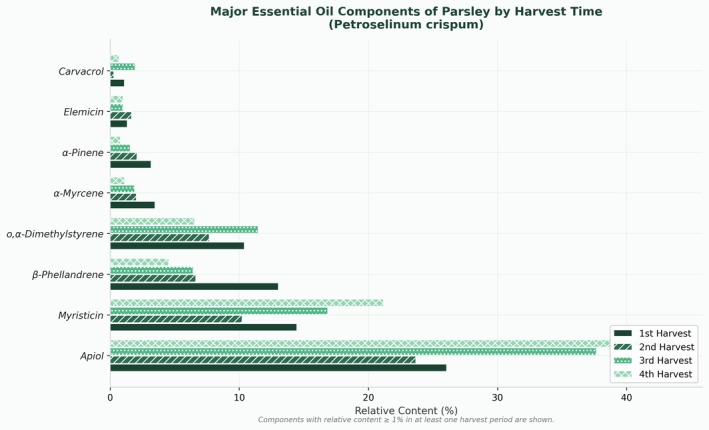
Relative content (%) of major essential oil components (≥ 1% in at least one harvest) in parsley ( *Petroselinum crispum* ) across four harvest times as determined by GC–MS analysis.

In trials conducted on the İzmir/Ödemiş ecotype, the EO content exhibited statistically significant differences depending on the harvest time (LSD: 0.019). These values, determined as 0.53% in the first harvest, 0.73% in the second, 0.23% in the third, and 0.40% in the fourth (Figure 2), maintained an average of 0.47% and demonstrated that EO accumulation is a dynamic process independent of plant biomass and highly sensitive to environmental conditions. Furthermore, three out of four harvest samples yielded EO content within the limits specified in the British Herbal Compendium (BHC) pharmaceutical quality monograph for parsley.

The obtained range of 0.23%–0.73% is in complete alignment with the general values of 0.1%–0.7% reported in the literature for parsley leaves (Melchior and Kastner 1974; Said‐Al Ahl et al. 2016; Subaş et al. 2024). The peak value of 0.7% reached in the second harvest represents the upper limit of this range and remains markedly higher than the ratios reported in different geographies such as Egypt (0.014%–0.173%) and Estonia (0.24%–0.29%) (Sany et al. 2022; Vokk et al. 2011; Alharbi et al. 2019). Similarly, Petropoulos et al. (2004) detected EO content in the range of 0.08%–0.30% in their study in Greece, whereas Gruszecki and Walasek‐Janusz (2022) reported values of 0.15%–0.45% in turnip‐rooted parsley cultivars in Poland (Petropoulos et al. 2004; Gruszecki and Walasek‐Janusz 2022). This comparison suggests that the ecological conditions of the Ödemiş region possess high potential for EO accumulation.

According to statistical analysis, the highest EO content was obtained in the second harvest (0.73%); however, in the fourth harvest, where the plant reached its maximum herbage yield, this ratio declined to 0.40%. This finding confirms that maximum biomass accumulation does not always coincide with maximum EO concentration and is consistent with similar downward trends reported by Said‐Al Ahl et al. (2016) and Sany et al. (2022). Petropoulos et al. (2004) also determined that EO content varies with the progression of the growth period and reported that higher EO ratios were achieved in early harvest stages. Unlike other agronomic parameters, the EO content exhibited a fluctuating pattern rather than a linear course, reflecting the dynamic transformation of the plant's metabolic priorities during the development process (Sany et al. 2022; Said‐Al Ahl et al. 2016).

#### 3.1.6 Essential Oil Composition

##### 3.1.6.1 General Profile

As a result of gas chromatography–mass spectrometry (GC–MS) analyses, a total of 55 compounds were identified in samples across four harvest periods; the identification rates were determined to be 94.47% for Pet‐1, 95.72% for Pet‐2, 96.47% for Pet‐3, and 96.02% for Pet‐4. These high identification rates indicate that the analytical quality remained at a reliable level throughout all harvest stages (Table 2; Figure 2). When the composition profile was examined, phenylpropanoids (apiol, myristicin, elemicin) were found to be the dominant group in all periods, followed by monoterpenes and sesquiterpenes.

**TABLE 2:** Average essential oil components of parsley ( *Petroselinum crispum* (Mill.) Fuss) for the experimental years.

No	RT	AI	Compound	% Pet‐1	% Pet‐2	% Pet‐3	% Pet‐4	ID
1	5.12	932	*α‐*Pinene	3.15	2.07	1.55	0.79	MS, AI
2	6.10	972	Sabinene	0.20	—	—	—	MS, AI
3	6.20	976	*β*‐Pinene	1.58	1.17	0.96	0.46	MS, AI
4	6.56	990	*α‐*Myrcene	3.46	2.01	1.88	1.11	MS, AI
5	6.97	1005	*α‐*Phellandrene	0.40	—	0.08	0.07	MS, AI
6	7.57	1023	*p*‐Cymene	1.60	1.13	1.24	0.59	MS, AI
7	7.79	1029	***β*‐Phellandrene**	**13.02**	**6.62**	**6.41**	4.54	MS, AI
8	8.72	1056	γ‐Terpinene	0.08	—	—	0.07	MS, AI
9	9.84	1089	***o*,*α‐*Dimethylstyrene**	**10.37**	**7.65**	**11.46**	**6.53**	MS, AI
10	10.17	1099	Linalool	0.07	—	0.07	0.07	MS, AI
11	10.62	1111	*p*‐Mentha‐1,3,8‐trien	1.05	—	—	0.05	MS, AI
12	10.99	1120	1,2‐methylenecycloheptanol	0.31	1.55	0.14	0.12	MS, AI
13	13.18	1175	Terpinene‐4‐ol	—	—	0.18	0.12	MS, AI
14	13.43	1182	*p*‐Acetyltoluene	—	0.83	1.15	0.79	MS, AI
15	13.50	1184	*p*‐Cymen‐8‐ol	0.83	1.32	1.10	0.87	MS, AI
16	13.72	1189	*α‐*Terpineol	1.34	—	0.13	0.08	MS, AI
17	14.20	1201	*o*‐Ethylanisole	0.37	0.24	0.29	0.14	MS
18	14.31	1204	1‐Methyl‐4‐(1‐methylpropyl)‐benzene	—	—	0.10	0.13	MS
19	14.61	1211	4,7‐Dimethyl‐benzofuran	0.11	—	0.28	0.32	MS
20	15.52	1233	Thymyl methylether	**—**	0.60	—	—	MS, AI
21	15.89	1242	Carvacrol methylether	0.15	0.19	—	0.18	MS, AI
22	17.14	1272	Phellandral	0.10	—	0.07	0.15	MS; AI
23	17.66	1284	Bornyl acetate	0.09	—	0.06	—	MS, AI
24	17.96	1292	Thymol	0.07	0.54	1.01	0.38	MS, AI
25	18.34	1301	Carvacrol	1.09	0.27	1.91	0.67	MS, AI
26	18.88	1314	2,6,6‐Trimethyl‐bicyclo(3,1,1)heptane‐2,3‐diol	0.17	0.54	—	—	MS
27	21.37	1374	***α‐*** Copaene	0.24	0.35	0.26	0.27	MS, AI
28	21.73	1383	2‐Methylisoborneol	1.71	3.73	0.67	0.42	MS
29	21.99	1389	*β*‐Elemene	—	1.25	—	—	MS, AI
30	22.10	1392	*β*‐Elemol	—	—	0.28	0.25	MS
31	22.35	1398	*4α‐*Methyl‐1,2,3,4,4α,5,6,7‐octahydro‐2‐naphtol	3.51	**7.76**	0.28	0.19	MS
32	22.47	1401	**α,α**,4‐Trimethyl‐benzenemethanol	0.50	1.79	0.32	*t*	MS
33	23.10	1417	Caryophyllene (Z)	1.06	3742	20.86	2.28	MS, AI
34	23.82	1435	3,7,7‐Trimethyl‐8‐(2‐methyl‐propenyl)‐bicyclo[4,2,0]oct‐2‐ene	0.35	2.56	0.28	0.14	MS
35	24.47	1451	*α‐*Humulene	—	*t*	0.09	0.16	MS, AI
36	24.68	1456	Caryophyllene (E)	0.14	0.27	0.14	0.09	MS
37	25.17	1468	Octahydro‐4‐hydroxy‐1(2H)‐naftalenone	0.57	2.41	0.36	0.14	MS, AI
38	25.59	1479	Germacrene‐D	0.97	*t*	0.52	0.45	MS, AI
39	25.70	1482	*p*‐Menthane‐1,2,3‐triol	—	4.43	—	—	MS
40	26.71	1507	*β*‐Bisabolene	0.17	0.26	0.32	0.18	MS, AI
41	27.32	1523	**Myristicin**	**14.44**	**10.21**	**16.85**	**21.15**	MS, AI
42	27.39	1525	Epiglobulol	—	0.59	0.82	1.04	MS, AI
43	28.54	1555	Viridiflorol	0.92	0.43	0.98	0.93	MS
44	28.61	1557	Elemicin	1.31	1.64	0.97	0.99	MS, AI
45	29.51	1580	Caryophyllene oxide	0.29	0.28	0.79	1.68	MS, AI
46	30.03	1593	Carotol	1.37	0.95	1.54	1.33	MS, AI
47	30.61	1609	2,4,4‐Trimethyl‐3‐[3‐oxobutyl]cyclohex‐2‐enone	0.24	0.25	0.16	0.20	MS
48	31.73	1640	*α‐*Cadinol	0.21	0.72	0.92	0.70	MS, AI
49	32.17	1652	*epi‐α‐*Cadinol	0.17	1.54	0.36	0.26	MS, AI
50	33.44	1686	**Apiol**	**26.05**	**23.66**	**37.65**	**43.69**	MS, AI
51	36.03	1760	Benzyl benzoate	0.25	—	0.24	0.32	MS, AI
52	38.69	1838	Neophytadiene	0.17	—	0.18	0.18	MS
53	38.88	1844	6,10,14‐Trimethyl‐2‐pentadecanone	—	—	—	0.14	MS, AI
54	42.76	1963	Hexadecanoic acid	—	—	0.25	0.26	MS, AI
55	47.31	2084	*trans*‐Phytol	0.22	0.17	0.31	0.35	MS, AI
			**Total %**	**94.47**	**95.72**	**96.47**	**96.02**	
			**Number of compounds**	**44**	**39**	**45**	**48**	
**Monoterpene hydrocarbons (%)**	**23.36**	**14.73**	**13.45**	**8.63**				
**Sesquiterpene hydrocarbons (%)**	**2.58**	**4.19**	**22.40**	**3.64**				

*Note:* The shaded cells indicate statistically distinct harvest groups based on significant differences among harvest times. The shading is used to visually distinguish between harvest periods for ease of comparison.

##### 3.1.6.2 Chemical Class Distribution

Identified components were categorized based on their chemical structures into monoterpenes, oxygenated monoterpenes, sesquiterpenes, oxygenated sesquiterpenes, phenylpropanoids, and other constituents (Table 3).

**TABLE 3:** Chemical class distribution (%) according to harvest periods.

Chemical class	Pet‐1	Pet‐2	Pet‐3	Pet‐4
Monoterpene hydrocarbons	23.36	14.73	13.45	8.63
Oxygenated monoterpenes	3.41	3.18	3.60	1.58
Sesquiterpene hydrocarbons	2.58	4.19	22.40	3.64
Oxygenated sesquiterpenes	3.27	2.86	3.28	3.36
Phenylpropanoids	41.80	35.51	55.47	65.83
F/M ratio^a^	1.79	2.41	4.12	7.63

^a^ F/M Ratio: Phenylpropanoid/Monoterpene ratio.

The total proportion of monoterpene hydrocarbons decreased progressively from Pet‐1 to Pet‐4 (23.36% → 8.63%, −63.1%). Conversely, the proportion of phenylpropanoid components increased significantly (41.80% → 65.83%, +57.5%). The phenylpropanoid/monoterpene (P/M) ratio rose from 1.79 to 7.63 during ontogenetic development (*R*2 = 0.89), which indicates a programmed shift in metabolic pathways. In Pet‐3, the proportion of sesquiterpene hydrocarbons was found to be abnormally high (22.40%), primarily due to the elevated concentration of caryophyllene (Z).

##### 3.1.6.3 Phenylpropanoids

Apiol was identified as the primary component of the composition profile in all harvest periods, with detection rates of 26.05% in Pet‐1, 23.66% in Pet‐2, 37.65% in Pet‐3, and 43.69% in Pet‐4. This increasing trend observed as the growth period advanced directly aligns with findings regarding the rise of apiol levels during ontogenetic development (Petropoulos et al. 2004). Gruszecki and Walasek‐Janusz (2022) reported apiol ratios in turnip‐rooted parsley cultivars ranging from 12% to 67%; this broad range emphasizes the decisive role of genotype × environment interactions on apiol accumulation (Gruszecki and Walasek‐Janusz 2022; Ruiz et al. 2022). Ruiz et al. (2022) reported apiol as the dominant constituent (61.94%) in organic leaf parsley EO, confirming the characteristic phenylpropanoid profile of this species. Furthermore, the high apiol values of the Ödemiş ecotype are in full alignment with research demonstrating that the apiol ratio is significantly higher in the leaf fraction compared to the stem (Sany et al. 2022).

Myristicin, the second dominant phenylpropanoid, was determined at ratios of 14.44% in Pet‐1, 10.21% in Pet‐2, 16.85% in Pet‐3, and 21.15% in Pet‐4. The value of 21.15% reached in the fourth harvest exceeds the 5%–18% range reported in Estonian studies (Vokk et al. 2011) and the 3%–15% range detected by Petropoulos et al. (2004) (Vokk et al. 2011; Petropoulos et al. 2004). In this context, research highlights the neurotoxic and hepatotoxic potential of myristicin, emphasizing that safety limits must be considered in the industrial application of essential oils with high myristicin content (Ozsoy‐Sacan et al. 2006). Consequently, monitoring the Pet‐4 sample for this parameter is critical for product safety.

This pattern is consistent with the quantity‐quality trade‐off discussed in section 4.1.5; the phenylpropanoid pathway (shikimate route) operates independently of the terpenoid pathway, enabling differential accumulation at different ontogenetic stages (Dewick 2009). Zheljazkov et al. (2008) demonstrated analogous harvest‐stage‐dependent shifts in terpenoid composition in basil ( *Ocimum basilicum* ), where delayed harvesting similarly favored the accumulation of heavier, more oxygenated secondary compounds at the expense of lighter monoterpenes, suggesting that this biosynthetic transition may be a broader feature of Apiaceae and Lamiaceae aromatic species.

##### 3.1.6.4 Monoterpenes

**FIGURE 3:**
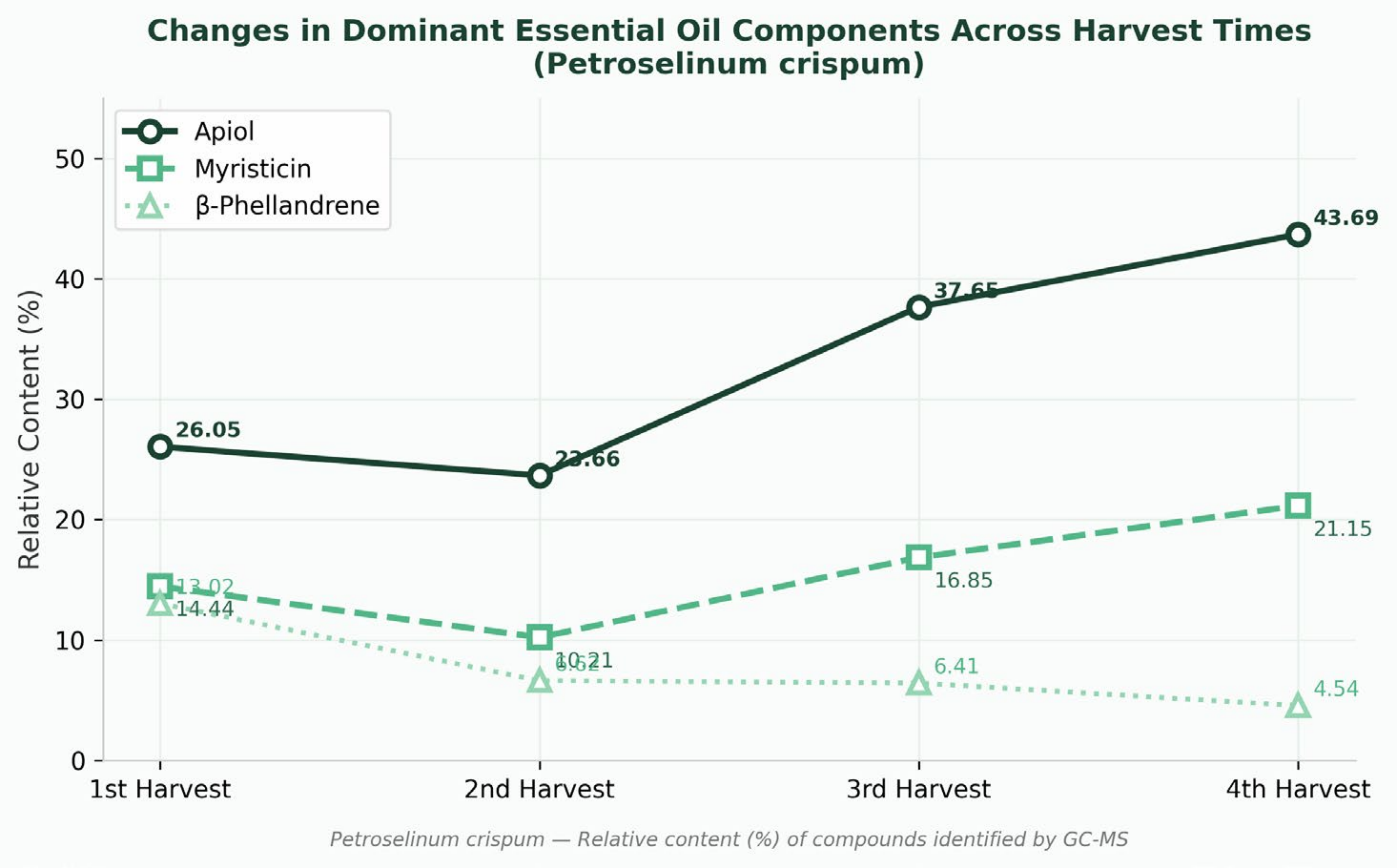
Changes in the relative content (%) of the three dominant essential oil components—Apiol, myristicin, and β‐phellandrene—across four harvest times in parsley ( *Petroselinum crispum* ).

In the monoterpene fraction, *β*‐phellandrene serves as a chemotaxonomic marker for parsley and remains a significant component across all developmental stages. Reaching its maximum value of 13.02% in Pet‐1, *β*‐phellandrene levels exhibited a continuous decline in subsequent periods, regressing to 4.54% in Pet‐4 (Figures 2 and 3). This downward trend aligns with the findings of Petropoulos et al. (2004), who noted that monoterpene ratios decrease as the growth period advances, and with reports by Sany et al. (2022) regarding the decisive influence of the harvest time × genotype interaction on *β*‐phellandrene. Furthermore, Gruszecki and Walasek‐Janusz (2022) reported *β*‐phellandrene ratios in the range of 3%–18% for various turnip‐rooted parsley cultivars; the Pet‐1 value for the Ödemiş ecotype is situated near the upper limit of this reported range. Mirmohammadmakki et al. (2023) similarly reported a progressive decline in β‐phellandrene across consecutive spring harvests of parsley, supporting the view that monoterpene biosynthesis is developmentally regulated and declines as phenylpropanoid pathways become dominant in later ontogenetic stages.

Other significant monoterpene components were identified as o,*α*‐dimethylstyrene (Pet‐1: 10.37%; Pet‐3: 11.46%), *α*‐myrcene (Pet‐1: 3.46%), and *α*‐pinene (Pet‐1: 3.15%). The high total monoterpene content detected in Pet‐2 (14.73%) provides evidence that monoterpene synthase enzymes reached their peak activity during this stage. While the 0.5% EO ratio in Pet‐1 represents a moderate production level during the early ontogenetic phase, the 0.7% peak value in Pet‐2 corresponds to the highest point of the plant's EO production capacity.

##### 3.1.6.5 Sesquiterpenes and Stress Response

**FIGURE 4:**
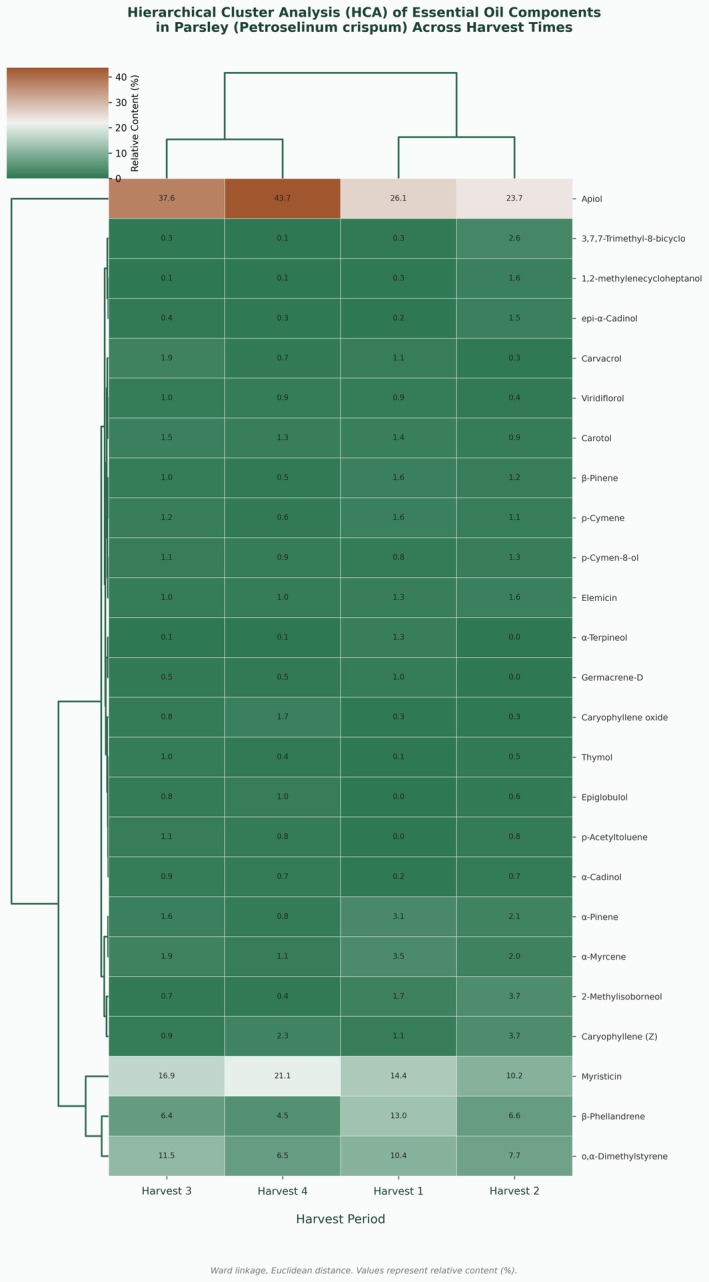
Hierarchical cluster analysis (HCA) heatmap of essential oil components in parsley ( *Petroselinum crispum* ) across four harvest times. Ward linkage, Euclidean distance. Cell values represent relative content (%). Color scale: Brown = high, green = low.

The most remarkable finding within the sesquiterpene fraction is the concentration of caryophyllene (Z), which surged to 20.86% in Pet‐3 (Figures 2 and 4). In the general literature, caryophyllene values for parsley typically range between 1% and 8%: Vokk et al. (2011) reported 0.31%, Gruszecki and Walasek‐Janusz (2022) noted 0.5%–3%, and Petropoulos et al. (2004) observed 0.3%–2%. The Pet‐3 value from the Ödemiş ecotype is approximately 3 to 20 times higher than these values, representing a level considerably higher than previously reported values for parsley leaf EO.

This marked increase cannot be attributed to ontogenetic shifts alone. It may be associated with environmental conditions prevalent at the time of the third harvest, such as high temperatures, although this explanation requires further controlled experimental validation. This may represent a possible defense‐like metabolic response triggered by high temperatures and potential water stress in July during the third harvest period (July 22). Caryophyllene is an energetically costly sesquiterpene whose biosynthesis is known to increase under heat and oxidative stress conditions (Bakkali et al. 2008; Niinemets 2010; Twaij and Hasan 2022). This indicates that the plant redirected its metabolic resources toward the synthesis of these complex structures during this stressful period (Gershenzon 1994). Indeed, the decline of caryophyllene to 2.28% in Pet‐4 consistent with the possibility as environmental conditions improved, metabolic priority may have shifted back toward phenylpropanoids.

This interpretation is consistent with the growth‐differentiation balance hypothesis, which posits resource competition between secondary metabolite synthesis and vegetative growth (Gershenzon 1994).

##### 3.1.6.6 Oxidative Compounds

The high proportions of oxidative monoterpene derivatives detected in the Pet‐2 sample—specifically 7.76% 4*α*‐Methyl‐1,2,3,4,4*α*,5,6,7‐octahydro‐2‐naphthol, 3.73% 2‐methylisoborneol, and 2.41% octahydro‐4‐hydroxy‐naphthalenone—indicate that the plant activated defense mechanisms against intense environmental stress during the second harvest period, experiencing a temporary surge in essential oil synthesis. These compounds can be evaluated as markers of oxidative stress and serve as early indicators of the defense response against reactive oxygen species (ROS). The decline of these values in Pet‐3 and Pet‐4 reflects either a reduction in oxidative stress or a complete shift in the plant's metabolic priorities. Zhang et al. (2006) established a direct relationship between parsley essential oil components and antioxidant capacity, reporting that high concentrations of these compounds enhance bioactive potential.

##### 3.1.6.7 Late‐Period Components: Diterpenes and Fatty Acids

The appearance of trans‐phytol (Pet‐4: 0.35%), neophytadiene (Pet‐3–4: 0.18%), and hexadecanoic acid (Pet‐3–4: 0.25%–0.26%) exclusively in the later harvest periods suggests that chlorophyll degradation and membrane lipid metabolism were activated during these stages. This finding supports the premise that the plant enters a senescence process during the Pet‐3 and Pet‐4 stages. This is consistent with the observations of Petropoulos et al. (2004), which state that the EO composition profile undergoes radical transformations as the growth period advances (Petropoulos et al. 2004; Figure 3).

##### 3.1.6.8 Harvest Strategy and Industrial Evaluation

The findings suggest that the harvest strategy must be determined according to the intended application. When maximum EO content is prioritized, early July (the second harvest) emerges as the most appropriate period. During this stage, both the EO content (0.7%) and the monoterpene fraction (primarily *β*‐phellandrene and *α*‐myrcene) reach their peak values. Conversely, early September (the fourth harvest) should be preferred when total biomass and apiol quantity are the primary objectives (Sany et al. 2022; Said‐Al Ahl et al. 2016). Although the EO content declines to 0.4% in early September, the concentrations of apiol (43.69%) and myristicin (21.15%) reach their maximum levels, and the total herbage yield increases significantly during this period (Said‐Al Ahl et al. 2016; Sany et al. 2022). It should be noted, however, that the EO content recorded at Pet‐4 (0.40%) falls below the minimum limit of 0.30% (v/m) specified by the British Herbal Compendium (BHC) for dried parsley herb, indicating that while Pet‐4 is optimal for biomass and apiol yield, it may not satisfy pharmacopoeial quality standards for EO content. In such cases, a blended or intermediate harvest strategy—combining Pet‐2 and Pet‐4 material—may offer a practical compromise between EO content and apiol concentration for herbal medicine applications.

Said‐Al Ahl et al. (2016) reported that phosphorus fertilization enhances EO content and compositional quality; this finding suggests that the high values observed in Ödemiş may partially stem from the regional soil mineral structure (Said‐Al Ahl et al. 2016). Furthermore, Kołota and Adamczewska‐Sowińska (2012) revealed that harvest date influences not only the EO profile but also the nitrate content, emphasizing the need for a multidimensional evaluation of harvest timing regarding food safety (Kołota and Adamczewska‐Sowińska 2012).

The elevated apiol‐myristicin concentrations and the notable caryophyllene accumulation at Pet‐3 suggest potential value for pharmaceutical and industrial applications, although broader comparative studies are needed for confirmation.

## 4 Discussion

### 4.1 Correlation Analysis Between Agronomic Parameters and Essential Oil Composition

Pearson correlations were calculated across the four harvest‐time means (*n* = 4, df = 2); the critical |*r*| value at *p* ≤ 0.05 (two‐tailed) is therefore 0.950, and results should be interpreted accordingly. Pearson correlation coefficients between agronomic parameters and key essential oil components are presented in Table 4. Correlation‐based findings indicate statistical associations only and should not be interpreted as evidence of causation; mechanistic confirmation requires controlled experimental approaches. The strong positive correlations observed between plant height and apiol concentration (*r* = +0.89, ns) and the F/M ratio (*r* = +0.95, *p* ≤ 0.05) indicate that vegetative growth and secondary metabolite accumulation are coordinated processes regulated in an integrated manner throughout ontogenetic development (Herms and Mattson 1992).

**TABLE 4:** Pearson correlation coefficients (r) between agronomic parameters and EO composition in parsley ( *Petroselinum crispum* ) across four harvest times.

Parameter	Apiol (%)	Myristicin (%)	β‐phellandrene (%)	F/M ratio
Plant height (cm)	+0.89 ns	+0.76 ns	−0.92 ns	+0.95*
Fresh herb yield (kg da^−1^)	+0.94 ns	+0.82 ns	−0.86 ns	+0.96*
Dry matter (%)	+0.98*	+0.91 ns	−0.95*	+0.99**
Drug herb yield (kg da^−1^)	+0.96*	+0.84 ns	−0.88 ns	+0.97*
Essential oil content (%)	−0.23 ns	−0.45 ns	+0.31 ns	−0.38 ns

*Note:* *n* = 4 (four harvest‐time means); critical *r* at *p* ≤ 0.05 (two‐tailed, df = 2) = 0.950.

Abbreviation: ns, not significant.

* *p* ≤ 0.05.

** *p* ≤ 0.01.

The positive correlation between fresh herb yield and apiol concentration (*r* = +0.94, ns) reveals a synergistic relationship between biomass accumulation and phenylpropanoid synthesis. This finding challenges the classical growth‐defense trade‐off hypothesis (Herms and Mattson 1992), suggesting that in late ontogenetic stages (Pet‐4), the plant is capable of simultaneously achieving maximum biomass production and maximum secondary metabolite accumulation. The strong positive correlation between dry matter content and the F/M ratio (r = +0.99, *p* ≤ 0.01) confirms that structural and chemical maturation progress in parallel.

Conversely, β‐phellandrene showed strong negative correlations with all biomass parameters (*r* = −0.86 to −0.95), of which only the correlation with dry matter (*r* = −0.95, *p* ≤ 0.05) reached statistical significance (critical *r* = 0.950, df = 2). The weak non‐significant negative correlation between EO content and apiol concentration (*r* = −0.23, ns) reflects a quantity‐quality trade‐off in which the plant distributes its limited metabolic resources across different secondary metabolite pathways (Herms and Mattson 1992; Gershenzon 1994). The pairing of peak oil content (Pet‐2: 0.7%) with relatively low apiol (23.66%) and reduced oil content (Pet‐4: 0.4%) with maximum apiol (43.69%) is consistent with this resource allocation strategy.

### 4.2 Principal Component Analysis

Principal component analysis (PCA) of EO composition effectively resolved all four harvest periods in two‐dimensional space, with PC1 and PC2 collectively accounting for 92.75% of total variance (Figure 6). The inclusion of the corrected Caryophyllene (Z) value for Pet‐3 (20.86%) was essential for the biological interpretation of the PCA structure.

PC1 explained 62.12% of total variance and was primarily driven by apiol (loading: +0.730) and myristicin (loading: +0.310), opposing β‐phellandrene (loading: −0.184) and monoterpene hydrocarbons. This axis represents the core ontogenetic metabolic shift from monoterpene‐dominated (Pet‐1, Pet‐2: negative PC1 scores) to phenylpropanoid‐dominant chemotypes (Pet‐3, Pet‐4: positive PC1 scores). Pet‐4 showed the highest positive PC1 score (+9.031), consistent with its maximum apiol concentration (43.69%).

PC2 explained 30.62% of variance and was dominated by Caryophyllene (Z) (loading: −0.840), clearly capturing the chemotypic distinctiveness of Pet‐3. The strongly negative PC2 score of Pet‐3 (−9.645) relative to all other harvest periods provides multivariate confirmation that the Pet‐3 sample represents a chemotypically distinct profile, driven by the notably high accumulation of caryophyllene (Z) at Pet‐3, possibly associated with prevailing high‐temperature conditions. Pet‐4, in contrast, showed the highest positive PC2 score (+10.881), reflecting its recovery to a phenylpropanoid‐dominant profile after the stress period subsided. The complete spatial separation of all four harvest periods in the biplot suggests that harvest timing is a major determinant of EO chemotype under the conditions of this study.

### 4.3 Chemotype Characterization

**FIGURE 5:**
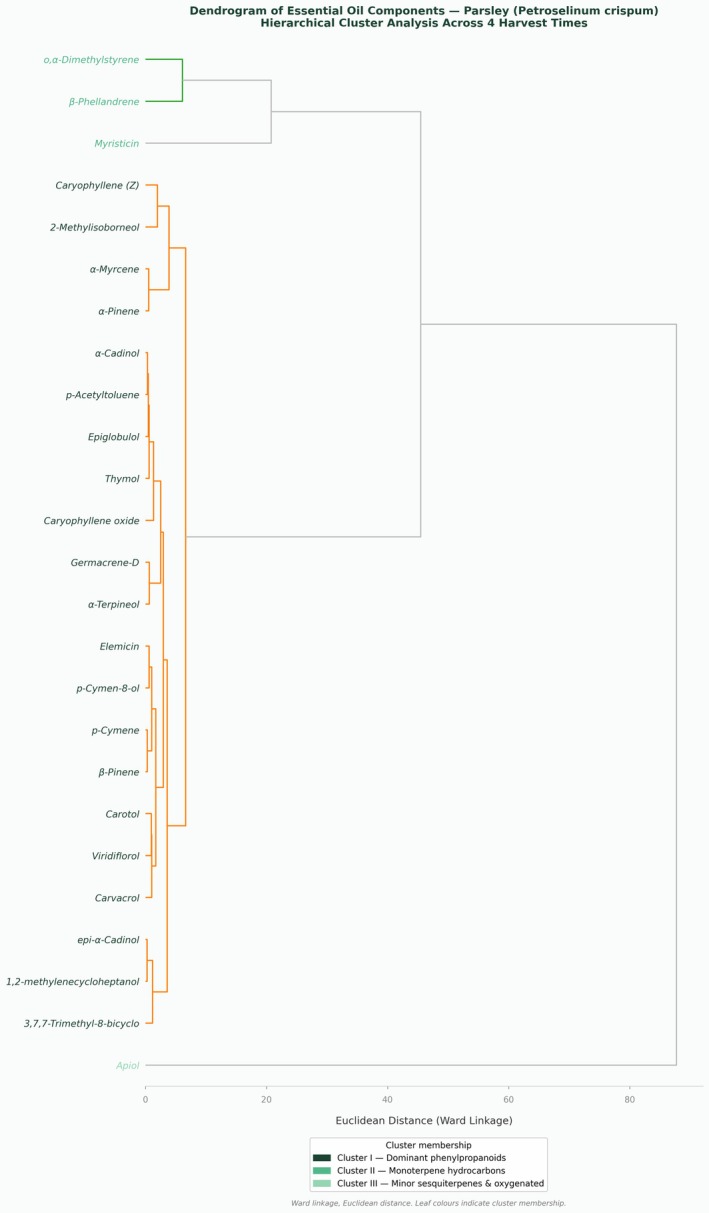
Dendrogram of essential oil components in parsley ( *Petroselinum crispum* ) based on hierarchical cluster analysis (Ward linkage, Euclidean distance) across four harvest times. Leaf colors indicate cluster membership: Cluster I = dominant phenylpropanoids; Cluster II = monoterpene hydrocarbons; Cluster III = minor sesquiterpenes and oxygenated compounds.

**FIGURE 6:**
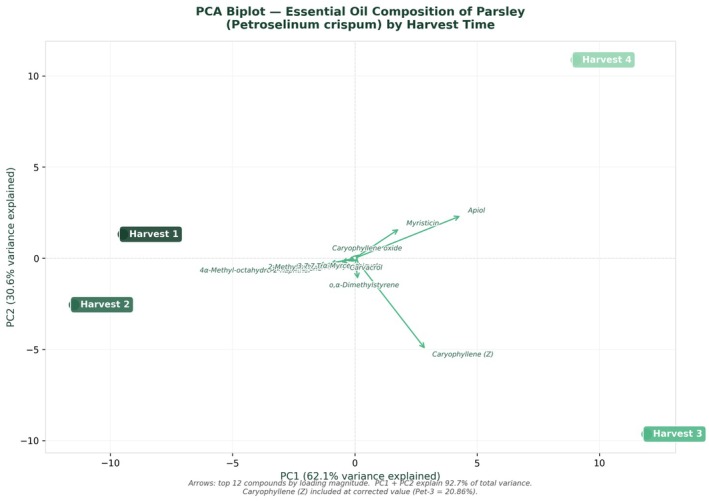
PCA biplot of EO composition in parsley ( *Petroselinum crispum* ) across four harvest times. Arrows indicate loading vectors of the top 12 compounds by loading magnitude. PC1 (62.1%) and PC2 (30.6%) together explain 92.7% of total variance. Caryophyllene (Z) included at corrected value (Pet‐3 = 20.86%).

Based on the integrated results of hierarchical cluster analysis and PCA (Figures 4, 5, 6), two distinct chemotypes were identified in the İzmir/Ȕdemiş parsley ecotype across the growing season (Table 5). Hierarchical cluster analysis further confirmed this separation, grouping Pet‐1 and Pet‐2 into one cluster and Pet‐3 and Pet‐4 into another based on compound loading patterns (Figure 5). This chemotypic differentiation reflects the progressive ontogenetic shift in secondary metabolite biosynthesis described throughout this study. It should be noted that the chemotype classification adopted here is operational, based on the relative proportions of major compound groups, following approaches used in the Apiaceae literature (Petropoulos et al. 2004; Gruszecki and Walasek‐Janusz 2022; Vokk et al. 2011; El‐Zaeddi et al. 2020; Figueiredo et al. 2008; Raal et al. 2012; Simon et al. 1990); formal chemotype designations would require broader genotypic sampling across multiple environments and years.

**TABLE 5:** Characteristic features of the two chemotypes identified in parsley ( *Petroselinum crispum* ) from the İzmir/Ödemiş ecotype.

Characteristic	Chemotype A	Chemotype B
Harvest periods	Pet‐1, Pet‐2	Pet‐3, Pet‐4
Phenylpropanoids (%)	35.51–41.80	55.47–65.83
Monoterpene hydrocarbons (%)	14.73–23.36	8.63–13.45
F/M ratio	1.79–2.41	4.12–7.63
Apiol (%)	23.66–26.05	37.65–43.69
Myristicin (%)	10.21–14.44	16.85–21.15
β‐phellandrene (%)	6.62–13.02	4.54–6.41
Marker compound	β‐phellandrene	Apiol

Chemotype A (Monoterpene‐Balanced Type) encompasses the early and mid‐season harvests (Pet‐1 and Pet‐2). This chemotype is characterized by a relatively higher proportion of monoterpene hydrocarbons (14.73%–23.36%) and a moderate phenylpropanoid content (35.51%–41.80%). The F/M ratio ranges from 1.79 to 2.41, and β‐phellandrene serves as the diagnostic marker compound. Essential oil content peaks within this chemotype (Pet‐2: 0.70%), making it the preferred harvest period when maximum oil yield per unit dry mass is the primary industrial objective.

Chemotype B (Phenylpropanoid‐Dominant Type) covers the late‐season harvests (Pet‐3 and Pet‐4). Phenylpropanoids dominate the oil profile (55.47%–65.83%), apiol reaches its maximum concentration (37.65%–43.69%), and the F/M ratio rises sharply to 4.12–7.63, representing a 2.3–3.2‐fold increase relative to Chemotype A. Apiol is the definitive marker compound of this chemotype. Pet‐3 within this group is notable for the transient induction of caryophyllene (Z) (20.86%), which may be associated with environmental conditions at the time of harvest, such as high summer temperatures, although controlled validation is required. Pet‐4 represents the commercially optimal variant of Chemotype B: maximum apiol content (43.69%), maximum biomass (10,076.33 kg da^−1^), and maximum drug herb yield (2170.89 kg da^−1^) converge in this single harvest.

The chemotypic transition observed in this study is consistent with the programmed biosynthetic shift reported for Apiaceae species during ontogenetic maturation (Petropoulos et al. 2004; Said‐Al Ahl et al. 2016; Raal et al. 2012). The clear separation of two chemotypes within a single ecotype across one growing season demonstrates that harvest timing effectively determines the pharmaceutical and industrial utility of parsley essential oil from the Ȕdemiş region. These findings provide a scientific framework for the standardization and targeted production of parsley‐derived ingredients according to end‐use requirements.

## 5 Conclusion

This study comprehensively demonstrated that harvest time is the primary determinant of both yield potential and EO chemotype in parsley ( *Petroselinum crispum* ) grown under the ecological conditions of İzmir/Ȕdemiş. The four harvest periods spanning late May to early September induced a progressive and statistically significant ontogenetic transition across all measured parameters.

From an agronomic perspective, the fourth harvest (early September) consistently produced the highest values for plant height (40.33 cm), fresh herb yield (10,076.33 kg da^−1^), dry matter content (20.17%), and drug herb yield (2170.89 kg da^−1^), representing a six‐fold increase in dry herb yield over the growing season. These values substantially exceed those reported for parsley cultivars under Egyptian, Greek, and Polish conditions, underscoring the high productive potential of the local Ȕdemiş ecotype.

Essential oil content followed a non‐linear pattern independent of biomass accumulation, peaking at the second harvest (0.73%, early July) and declining to a minimum at the third harvest (0.23%, late July). This decoupling reflects a quantity‐quality trade‐off in which limited metabolic resources are redistributed among competing biosynthetic pathways at different ontogenetic stages.

GC–MS analysis identified 55 volatile compounds across the four harvest periods, with overall identification rates of 94.47%–96.47%. The essential oil was characterized as an apiol/myristicin phenylpropanoid chemotype throughout all stages. A progressive biosynthetic shift from monoterpene‐dominated (Chemotype A: Pet‐1, Pet‐2) to phenylpropanoid‐dominant (Chemotype B: Pet‐3, Pet‐4) composition was confirmed by correlation analysis, HCA, PCA, and chemotype characterization. The F/M ratio increased from 1.79 to 7.63 over the growing season (*R* ^2^ = 0.89), consistent with a developmentally regulated metabolic transition.

A particularly noteworthy finding was the notably high accumulation of caryophyllene (Z) at the third harvest (20.86%), a value considerably exceeding typical reported ranges in the parsley leaf essential oil literature. This accumulation may be associated with environmental conditions at the time of harvest, such as high temperatures and potential water stress in late July; this interpretation is supported by PCA, in which Pet‐3 exhibited a strongly negative PC2 score driven by the caryophyllene (Z) loading. The concurrent reduction in total EO content at this harvest (0.23%) further supports the hypothesis of resource redirection toward energetically costly sesquiterpene biosynthesis under stress.

The observed variation in essential oil composition, particularly the increase in compounds such as caryophyllene and myristicin, may have implications for the biological activity and functional value of parsley essential oil, as these compounds have been reported to exhibit antioxidant and antimicrobial properties (Bakkali et al. 2008; Zhang et al. 2006).

From a practical standpoint, the findings indicate that the optimal harvest strategy must be aligned with the intended end use: early July (Pet‐2) should be preferred when maximum EO content and a monoterpene‐rich profile are required, while early September (Pet‐4) is optimal for maximum biomass, maximum apiol concentration, and highest drug herb yield. The results of this study should be interpreted in light of its inherent limitations: data were obtained from a single growing year, a single field location (Ödemiş/İzmir), and a single local ecotype. Multi‐year replicated trials and multi‐location comparisons with other parsley genotypes are necessary to confirm the generalisability of the agronomic and chemotypic patterns reported here. Subject to such validation, the Ödemiş ecotype shows potential as a valuable genetic resource for pharmaceutical and industrial applications, and the harvest‐period framework developed in this study may serve as a basis for further investigation through controlled field experiments and targeted breeding programmes. Future studies conducted across multiple environments and growing seasons are required to validate the stability and generalisability of these findings. These findings may contribute to optimizing harvest strategies for both yield and functional quality of parsley essential oil.

## Author Contributions

**Bintuğ Öztürk:** methodology, software, validation. **Ayşe Betül Avci:** writing – original draft, project administration, supervision, formal analysis, conceptualization. **Özlem Alan:** data curation, investigation. **Merve Göre:** writing – review and editing, visualization, formal analysis.

## Conflicts of Interest

The authors declare no conflicts of interest.

## Supporting information


**Figure S1:** Comparison of GC–MS analysis profiles obtained from the third injections of each of the four research samples.

## Data Availability

The data that support the findings of this study are available from the corresponding author upon reasonable request.

## References

[fsn372235-bib-0001] Abdalla, A. I. , and S. A. W. Elriah . 2024. “Characterization, Yield, Growth, and Essential Oil of Parsley Local Lines of Sudan.” Ricos Biology Journal 2, no. 2: 71–85.

[fsn372235-bib-0002] Adams, R. P. 2007. Identification of Essential Oil Components by Gas Chromatography/Mass Spectrometry. 4th ed. Allured Publishing Corporation.

[fsn372235-bib-0003] Ahmed, W. E. , A. A. Almutairi , M. S. Almujaydil , R. Algonaiman , H. M. Mousa , and R. M. Alhomaid . 2025. “Nutraceutical Potential of Parsley ( *Petroselinum crispum* Mill.): Comprehensive Overview.” Italian Journal of Food Science 37, no. 1: 194–209. 10.15586/ijfs.v37i1.2806.

[fsn372235-bib-0004] Alharbi, B. M. , A. A. Mahmoud , T. Astatkie , and H. A. H. Said‐Al Ahl . 2019. “Growth and EO Composition Responses of Parsley Cultivars to Phosphorus Fertilization and Harvest Date.” Journal of Plant Nutrition 42, no. 18: 2395–2405. 10.1080/01904167.2019.1656243.

[fsn372235-bib-0005] Antonopoulos, A. , I. C. Karapanos , S. A. Petropoulos , and H. C. Passam . 2014. “The Effect of Two Levels of Ammonium Nitrate Application on the Yield of Plain‐Leaf, Curly‐Leaf and Turnip‐Rooted Parsley and the Quality and EO Composition of the Leaves Before and After Storage in a Partially Dehydrated Form.” Analele Universităţii Din Oradea, Fascicula Biologie 21, no. 2: 65–69.

[fsn372235-bib-0006] Aziz, E. E. , R. M. Sabry , and S. S. Ahmed . 2013. “Plant Growth and Essential Oil Production of Sage ( *Salvia officinalis* L.) and Curly‐Leafed Parsley ( *Petroselinum crispum* Ssp. *Crispum* L.) Cultivated Under Salt Stress Conditions.” World Applied Sciences Journal 28, no. 6: 785–796.

[fsn372235-bib-0007] Bahramsoltani, R. , R. Ahmadian , M. Daglia , and R. Rahimi . 2024. “ *Petroselinum crispum* (Mill.) Fuss (Parsley): An Updated Review of the Traditional Uses, Phytochemistry, and Pharmacology.” Journal of Agricultural and Food Chemistry 72, no. 2: 956–972. 10.1021/acs.jafc.3c06429. 38189231

[fsn372235-bib-0008] Bakkali, F. , S. Averbeck , D. Averbeck , and M. Idaomar . 2008. “Biological Effects of Essential Oils: A Review.” Food and Chemical Toxicology 46, no. 2: 446–475. 10.1016/j.fct.2007.09.106. 17996351

[fsn372235-bib-0009] Borges, I. B. , B. K. Cardoso , E. S. Silva , et al. 2016. “Evaluation of Performance and Chemical Composition of *Petroselinum crispum* Essential Oil Under Different Conditions of Water Deficit.” African Journal of Agricultural Research 11, no. 6: 480–486. 10.5897/AJAR2015.10748.

[fsn372235-bib-0010] Dewick, P. M. 2009. Medicinal Natural Products: A Biosynthetic Approach. 3rd ed. John Wiley & Sons. 10.1002/9780470742761.

[fsn372235-bib-0011] El‐Zaeddi, H. , Á. Calín‐Sánchez , L. Noguera‐Artiaga , J. Martínez‐Tomé , and Á. A. Carbonell‐Barrachina . 2020. “Optimization of Harvest Date According to the Volatile Composition of Mediterranean Aromatic Herbs at Different Vegetative Stages.” Scientia Horticulturae 267: 109336. 10.1016/j.scienta.2020.109336.

[fsn372235-bib-0012] Figueiredo, A. C. , J. G. Barroso , L. G. Pedro , and J. J. C. Scheffer . 2008. “Factors Affecting Secondary Metabolite Production in Plants: Volatile Components and Essential Oils.” Flavour and Fragrance Journal 23, no. 4: 213–226. 10.1002/ffj.1875.

[fsn372235-bib-0013] Gershenzon, J. 1994. “Metabolic Costs of Terpenoid Accumulation in Higher Plants.” Journal of Chemical Ecology 20, no. 6: 1281–1328. 10.1007/BF02059810. 24242341

[fsn372235-bib-0015] Gruszecki, R. , and A. Sałata . 2013. “Effect of Sowing Date on Biometrical Features of Hamburg Parsley Plants.” Modern Phytomorphology 3: 125–129.

[fsn372235-bib-0016] Gruszecki, R. , and M. Walasek‐Janusz . 2022. “Essential Oil Diversity of Turnip‐Rooted Parsley Cultivars.” Agronomy 12, no. 8: 1949. 10.3390/agronomy12081949.

[fsn372235-bib-0017] Herms, D. A. , and W. J. Mattson . 1992. “The Dilemma of Plants: To Grow or Defend.” Quarterly Review of Biology 67, no. 3: 283–335. 10.1086/417659.

[fsn372235-bib-0018] Kołota, E. , and K. Adamczewska‐Sowińska . 2012. “The Effects of Genetic and Agronomic Factors on Quantity and Quality of Leafy Parsley Yield.” Polish Journal of Environmental Studies 21, no. 4: 937–942.

[fsn372235-bib-0019] Lopez, M. G. , I. R. Sanchez‐Mendoza , and N. Ochoa‐Alejo . 1999. “Comparative Study of Volatile Components and Fatty Acids of Plants and in Vitro Cultures of Parsley ( *Petroselinum crispum* (Mill.) Nym. Ex Hill).” Journal of Agricultural and Food Chemistry 47, no. 8: 3292–3296. 10.1021/jf981159o. 10552648

[fsn372235-bib-0020] Melchior, H. , and H. Kastner . 1974. Gewürze: Botanische und chemische Untersuchung. Verlag Paul Parey.

[fsn372235-bib-0021] Mirmohammadmakki, F. , M. Gharachorloo , M. Ghavami , V. Abdossi , and R. Azizinezhad . 2023. “Quantitative Changes in Essential Oils Contents of Parsley ( *Petroselinum crispum* ) Harvested in Three Consecutive Months of Spring.” Journal of Food and Bioprocess Engineering 6, no. 2: 48–55.

[fsn372235-bib-0022] Niinemets, Ü. 2010. “Mild Versus Severe Stress and BVOCs: Thresholds, Priming and Consequences.” Trends in Plant Science 15, no. 3: 145–153. 10.1016/j.tplants.2009.11.008. 20006534

[fsn372235-bib-0023] Nouioura, G. , M. El Fadili , N. El Hachlafi , et al. 2024. “ *Petroselinum crispum* L., Essential Oil as Promising Source of Bioactive Compounds, Antioxidant, Antimicrobial Activities: In Vitro and in Silico Predictions.” Heliyon 10, no. 8: e29520. 10.1016/j.heliyon.2024.e29520. 38660278 PMC11040043

[fsn372235-bib-0024] Ozsoy‐Sacan, O. , R. Yanardag , H. Orak , Y. Ozgey , A. Yarat , and T. Tunali . 2006. “Effects of Parsley ( *Petroselinum crispum* ) Extract Versus *S*‐Methylisothiourea on Ileal Injury of Rats With Experimentally Induced Type 2 Diabetes.” Journal of Ethnopharmacology 104, no. 1–2: 175–181. 10.1016/j.jep.2005.08.067. 16223573

[fsn372235-bib-0025] Petropoulos, S. A. , D. Daferera , C. A. Akoumianakis , H. C. Passam , and M. G. Polissiou . 2004. “The Effect of Sowing Date and Growth Stage on the EO Composition of Three Types of Parsley ( *Petroselinum crispum* ).” Journal of the Science of Food and Agriculture 84, no. 12: 1606–1610. 10.1002/jsfa.1846.

[fsn372235-bib-0026] Raal, A. , E. Arak , and A. Orav . 2012. “The Content and Composition of the Essential Oil of *Petroselinum crispum* (Mill.) Fuss From Estonia.” Journal of Essential Oil Research 24, no. 5: 443–449. 10.1080/10412905.2012.703530.

[fsn372235-bib-0027] Ruiz, S. P. , S. S. Mendes , C. Frederico , et al. 2022. “Parsley ( *Petroselinum crispum* ): Chemical Composition and Antibacterial Activity of Essential Oil From Organic Against Foodborne Pathogens.” African Journal of Agricultural Research 16, no. 5: 605–611.

[fsn372235-bib-0028] Said‐Al Ahl, H. A. H. , M. Abou‐Ellail , and E. A. Omer . 2016. “Harvest Date and Genotype Influences Growth Characters and Essential Oil Production and Composition of *Petroselinum crispum* Plants.” Journal of Chemical and Pharmaceutical Research 8, no. 5: 992–1003.

[fsn372235-bib-0029] Sany, H. , H. A. H. Said‐Al Ahl , and T. Astatkie . 2022. “Essential Oil Content, Yield, and Components From the Herb, Leaf, and Stem of Curly‐Leafed Parsley at Three Harvest Days.” Journal of Central European Agriculture 23, no. 1: 54–61. 10.5513/JCEA01/23.1.3364.

[fsn372235-bib-0030] Shamshiri, M. , M. Ayyari , A. Mokhtassi‐Bidgoli , and M. Shams‐Bakhsh . 2024. “Effect of Viral‐Inoculation on the Morphology, Physiology, and Phytochemistry of Parsley ( *Petroselinum crispum* ).” Physiological and Molecular Plant Pathology 134: 102477.

[fsn372235-bib-0031] Simon, J. E. , A. F. Chadwick , and L. E. Craker . 1990. Herbs: An Indexed Bibliography 1971–1980. The Scientific Literature on Selected Herbs and Aromatic and Medicinal Plants of the Temperate Zone. Archon Books.

[fsn372235-bib-0032] Subaş, T. , U. Özgen , İ. Gökkaya , and G. Renda . 2024. “ *Petroselinum crispum* (Mill.) Fuss (Parsley), a Food and Medicinally Important Plant: A Review of Recent Studies Between 2013‐2023.” Journal of Faculty of Pharmacy of Ankara University 48, no. 2: 727–750. 10.33483/jfpau.1362626.

[fsn372235-bib-0033] Twaij, B. M. , and M. N. Hasan . 2022. “Understanding the Consequence of Environmental Stress for Accumulation of Secondary Metabolites in Medicinal and Aromatic Plants.” Journal of Applied Research on Medicinal and Aromatic Plants 28: 100362. 10.1016/j.jarmap.2022.100362.

[fsn372235-bib-0034] Ulukanli, Z. , S. Karabörklü , F. Bozok , et al. 2014. “Chemical Composition, Antimicrobial, Insecticidal, Phytotoxic and Antioxidant Activities of Mediterranean Pinus Brutia and *Pinus pinea* Resin Essential Oils.” Chinese Journal of Natural Medicines 12, no. 12: 901–910. 10.1016/S1875-5364(14)60135-9. 25556061

[fsn372235-bib-0035] Vokk, R. , T. Lõugas , K. Mets , and M. Kravets . 2011. “Dill ( *Anethum graveolens* L.) and Parsley ( *Petroselinum crispum* (Mill.) Fuss) From Estonia: Seasonal Differences in Essential Oil Composition.” Agronomy Research 9, no. Special Issue II: 515–520.

[fsn372235-bib-0036] Wichtl, M. 2004. Herbal Drugs and Phytopharmaceuticals: A Handbook for Practice on a Scientific Basis. 3rd ed. Medpharm Scientific Publishers, CRC Press.

[fsn372235-bib-0037] Zhang, H. , F. Chen , X. Wang , and H.‐Y. Yao . 2006. “Evaluation of Antioxidant Activity of Parsley ( *Petroselinum crispum* ) Essential Oil and Identification of Its Antioxidant Constituents.” Food Research International 39, no. 8: 833–839. 10.1016/j.foodres.2006.03.007.

[fsn372235-bib-0038] Zheljazkov, V. D. , C. L. Cantrell , B. Tekwani , and S. I. Khan . 2008. “Content, Composition, and Bioactivity of the Essential Oils of Three Basil Genotypes as a Function of Harvesting.” Journal of Agricultural and Food Chemistry 56, no. 2: 380–385. 10.1021/jf071948s. 18095647

