# Supplementary material for: Harvest Stage‐Dependent Variation in Essential Oil Chemotype and Agronomic Yield of Parsley (Petroselinum crispum (Mill.) Fuss)
Source: Food Sci Nutr. 2026 Aug 7;14(8):e72235. doi: 10.1002/fsn3.72235 (PMC13451938; doi:10.1002/fsn3.72235)
Supplement: Supplementary file 1 [file FSN3-14-e72235-s001.docx]

"Supplementary Figure S1" Comparison of GC-MS analysis profiles obtained from the third injections of each of the four research samples.
